# Do first responders and populations perceive risks similarly? A comparative study of seven countries

**DOI:** 10.3389/fpsyg.2023.1219927

**Published:** 2024-01-11

**Authors:** Nathan Stolero, Sahar Elkady, Leire Labaka, Maya Siman Tov, Kobi Peleg, Bruria Adini

**Affiliations:** ^1^Department of Emergency and Disaster Management, School of Public Health, Faculty of Medicine, Tel Aviv University, Tel Aviv, Israel; ^2^TECNUN—University of Navarra, San Sebastian, Spain; ^3^Magen David Adom, Tel Aviv, Israel; ^4^Israel Academic College, Ramat Gan, Israel; ^5^ResWell Research Collaboration on Resilience and Well-Being, Tel Aviv University, Tel Aviv, Israel

**Keywords:** risk perception, resilience, pandemics, nature-related events, extreme weather, critical services dependencies, social disruptions

## Abstract

**Introduction:**

Risk perception illustrates the subjective evaluation of individuals concerning the characteristics, severity, and capacity to cope with potential hazards. Risk perception influences attitudes and actions individuals take to protect themselves from future threats. Risk perceptions might change among different stakeholder groups such as society and first responders. Identifying risk perceptions of stakeholders is essential to establish effective protective measures.

**Method:**

This study investigated the commonalities and diversities in risk perception among first responders and the public, within and between seven European and beyond countries. A self-administered questionnaire was used to gather data from both first responders and civilians. They were asked to assess their risk perception level for five categories of risks (Extreme weather-related events, nature-related events, social disruptions, critical services dependencies, and pandemics).

**Results:**

Using Univariate Analysis of Variance showed disparity concerning both the levels of risk perception between the public and first responders, as well as their relative ranking. For example, concerning extreme weather-related and nature-related events, risk perception levels of the first responders is higher than that of the population in six out of the seven studied countries. In contrast, the population’s risk perception is higher compared to the first responders in six out of the seven countries, concerning critical infrastructure dependencies and pandemics.

**Discussion:**

The relative gaps between the first responders versus the population, within each country, vary considerably. Norway for example presents significant differences between the two internal populations concerning all risks (except for extreme weather), while in Sweden, no significant gaps were identified, concerning all five risks.

## Introduction

All societies are exposed to numerous risks that pose a threat to the well-being of their population, due to natural or human-made occurrences. Pandemics, floods, earthquakes, wars, industrial collapses, and more, frequently occur worldwide, impacting the safety and security of many communities. Preparing for, coping with, and overcoming such risks are highly dependent on the resilience of societies, which is determined, as shown by Bodas et al. (2022), by levels of trust, individual resilience, individual preparedness, and risk awareness, among other factors. In this article, we examine the gaps in risk perceptions of emergency professionals and first responders compared to those of the general population. Gaps, which as we present in the article, can affect essential factors of societal resilience, and thus may impact on the capacity of societies to react to such risks.

Risk perception is the subjective judgment that individuals make concerning the attributes, severity, and means of coping with various hazards (Grima et al., 2021). It reflects the appraisal of people concerning the likelihood of the danger and its potential adverse consequences (Bubeck and Botzen, 2013; Lechowska, 2018). Risk perceptions pertain to both the perceived severity of the situation (the potential damage that may incur), as well as the perceived vulnerability (probability of being negatively impacted) of oneself or that of loved ones (Kollmann et al., 2022).

Risk perception significantly influences various aspects of public preparedness for and function during emergencies. Risk perception was found to be associated with knowledge and information about appropriate actions in different emergency situations, adherence to recommendations and instructions, and communication with official emergency authorities (Bodas et al., 2022). Simultaneously, risk perception plays a critical role in the context of emergency professionals and first responders responsible for managing emergencies and disasters and was found to be correlated with factors such as motivation (Elkady et al., 2022). Consequently, discrepancies in risk perceptions concerning various hazards between first responders and the general population may undermine societal resilience. For instance, such gaps may diminish trust levels if the public feels that their concerns about perceived risks are not adequately addressed by first responders. Additionally, these gaps may reduce individual and public preparedness for threats that are perceived as less risky by the general population compared to first responders. Subsequently, this article aims to identify these disparities and emphasize similarities that may bolster societal resilience. Diverse behavioral models explain the variability in risk perceptions of different populations (Turner et al., 2006; Rudisill, 2013). For example, the psychometric model focuses mainly on the psychological management of human thoughts, decision-making, and subsequently – implementation of actions (Kiani et al., 2022), while the cultural model centers on the cognitive processes that impact thoughts and beliefs that lead to any measures that are adopted (Rippl, 2002). The Health Belief Model (Kamran et al., 2021) and the Protection Motivation Theory (Gumasing et al., 2022) posit that people will be more highly inclined to adopt both beliefs and behaviors when they consider a situation to be more severe (potentially detrimental) and themselves more vulnerable to its effects (Trifiletti et al., 2022).

Different risk perceptions may stem from varied factors including demographic characteristics (such as age, gender, and socio-economic status) (Brown et al., 2021; Kollmann et al., 2022; Shah et al., 2022); personality traits (such as ways of coping with stressful situations, views concerning fate versus control of events; leadership qualities) (Al-Dahash et al., 2022); cultural and social contexts (for example, local values and norms, or trust in data and in the authorities) (Renn and Rohrmann, 2000; Cori et al., 2022); assorted beliefs (such as religion, level of religiosity, fears, political or other attitudes) (Grima et al., 2021; Siegrist et al., 2021); as well as familiarity or knowledge about the hazard (Al-Dahash et al., 2022). The Social Amplification of Risk Framework (SARF) suggests that the interaction between psychological, cultural, social, and contextual factors, and the characteristics of the adversities, impact the risk perception and consequently, also influence protective behavior (Knuth et al., 2014).

Risk perceptions must be taken into consideration by risk managers, as they affect both attitudes and actions of the population (Lechowska, 2018). Risk perceptions have been found as significant predictors of health-related protective behaviors (Floyd et al., 2000; Scovell et al., 2022), though there is controversy concerning their relative impact. Several studies have shown that risk perceptions are only weakly or not at all associated with personal behavior that aims to protect the individual from adversity (Bubeck and Botzen, 2013; Lindell, 2013). In contrast, other studies have shown that risk perceptions positively impact protective behavior and contribute toward the adoption of measures that are vital to increasing the safety and resilience of populations (Scovell et al., 2022). It has been claimed that people tend to adopt protective (and preventive) measures when they believe that either they or others close to them may be negatively impacted by the different hazards (Kahlor et al., 2006; Dryhurst et al., 2020; Harper et al., 2020). Several studies have presented that people with higher risk perceptions expressed higher levels of compliance with protective behavior that was recommended (Barr et al., 2008; Jacobs et al., 2010). It should though be noted that there may be discrepancies between the intention to comply with recommended protective behavior and the actual adherence to such behavioral measures, otherwise known as the intention-behavior gap (Park et al., 2021; Kollmann et al., 2022).

Emerging from the classic theory of risk perceptions, scholars introduced the risk perception paradox, a phenomenon that challenges the conventional understanding of how individuals respond to perceived risks. While it’s commonly believed that a high risk perception would naturally lead to personal preparedness and subsequent risk mitigation behaviors, the reality is more complex. Studies have shown that even when individuals possess a high awareness of risks, they might not necessarily take appropriate preparedness actions (Shapira et al., 2018). This paradoxical behavior can be attributed to various factors. Firstly, individuals might recognize the risk but choose to accept it, especially if the perceived benefits, such as residing near a river, outweigh the potential hazards. Secondly, while individuals might understand the risk, they may not feel empowered to act, often transferring the responsibility to others. Lastly, there are instances where individuals, despite understanding the risk, might lack the resources, both economic and personal, to make meaningful changes. This intricate relationship between risk perception and actual preparedness actions underscores the need for a nuanced approach in risk governance and communication (Wachinger et al., 2013).

In response to any adversity, authorities and first responders must communicate with the population, to encourage the adoption of protective behavior by all individuals, to ensure their safety and survivability. The risk perception of both sectors (authorities/first responders versus the civil society) is vital to enhance effective preparedness and response to the situation. Nonetheless, it cannot be assumed that these two different groups in society similarly perceive the risk. Authorities and first responders need to recognize the similarities and differences that may prevail in their risk perceptions compared to that of the population. Many studies have been conducted among either first responders or varied populations (Lachlan et al., 2021; Spett, 2021; Cuesta et al., 2022). Furthermore, Elkady et al. (2022) identified the needs of first responders from the public to better manage any adversity. In contrast, despite an extensive literature review, no studies were found that compared the risk perceptions of first responders with those of civil society members.

Risk perceptions have also been found to differ among varied societies, even when they face similar threats. For example, despite the comparable risk for terror events among European countries, a relatively higher level of risk perception was identified over time in specific countries, such as England, Spain, and Turkey (Drakos and Müller, 2014), while concerning nuclear threat, French people perceived the risk as highest, compared to British, Spanish, and Swedish individuals (Viklund, 2003). Knuth et al. (2014) identified different levels of risk perception concerning earthquakes as well as other hazards (such as fires, floods, or terror events) in seven European countries (Germany, the Czech Republic, Italy, Turkey, Spain, Sweden, and Poland). Similarly, significant variability in risk perception, distress levels, and perceived readiness was reported during the COVID−19 pandemic among medical responders, such as among physicians from Spain, Belgium, and France (Guerrisi et al., 2022). Similar variability was identified concerning risk perceptions of local populations among eight different European countries, in a study that was conducted during the COVID−19 pandemic; although the individual respondents from the eight societies all ranked the pandemic as being the highest risk (out of five potential risks, including social disruptions, extreme weather, pandemic, critical services dependencies, and, nature-related events), the relative severity and probability of the risks varied among the respective societies (Bodas et al., 2022). The same dataset of that study is used in the current study.

Considering the importance of better understanding the realm of risk perceptions, the aim of the study was to identify commonalities and diversities in risk perceptions between first responders and civil populations among and between seven countries, within and beyond Europe.

## Methods

The study was cross-sectional, whence the data collection was conducted simultaneously in seven countries, within and beyond Europe. The primary method used for data collection was a self-administered questionnaire. Questionnaires are an effective technique to gather data from large samples as they provide a standardized set of questions that are easily interpretable by all the responders (Saunders et al., 2009). The uniform set of responses allows for a robust quantitative analysis of the results.

### Study population and sampling

The study investigated the risk perceptions of two different types of populations: the emergency responders and authorities (Group 1), and the civilians (Group 2) in seven countries: France, Israel, Italy, Norway, Romania, Spain, and Sweden. These countries differ in the characteristics of their populations as they cover both Western and Eastern European countries as well as one country outside of Europe. The study was conducted in January and February 2021, amid the COVID−19 pandemic.

For Population number 1 we targeted first responders from organizations such as the civil defense, firefighters, police, medical staff, NGOs, or governmental authorities who are experienced in dealing with emergencies. Regarding Population number 2, we targeted civilians over the age of 18. At least 500 respondents, representing the various groups of the population in each country, were recruited. Stratified sampling was used to ensure the inclusion of the varied groups, based on the Central Bureau of Statistics in the respective countries, considering age, gender, and geographic location.

### Study tools

The study tools were quantitative, internet-based questionnaires that were used to assess the risk perceptions of emergency responders and authorities (Population no. 1) and civilians (Population no. 2) for five different categories of risks as defined by UNESCO as follows (Rohit et al., 2010):

- Extreme weather-related events (e.g., cyclones, flooding, snow, droughts, wildfires),
- Nature-related events (e.g., geophysical events, earthquakes, tsunamis, landslides, volcanoes),
- Social disruptions (e.g., technological events, cyber-attacks, terrorist attacks, protests, riots, massive human displacements),
- Critical services dependencies (e.g., transportation networks, water, and energy.),
- Pandemics (e.g., biological events, contagious diseases).

**Figure 1:**
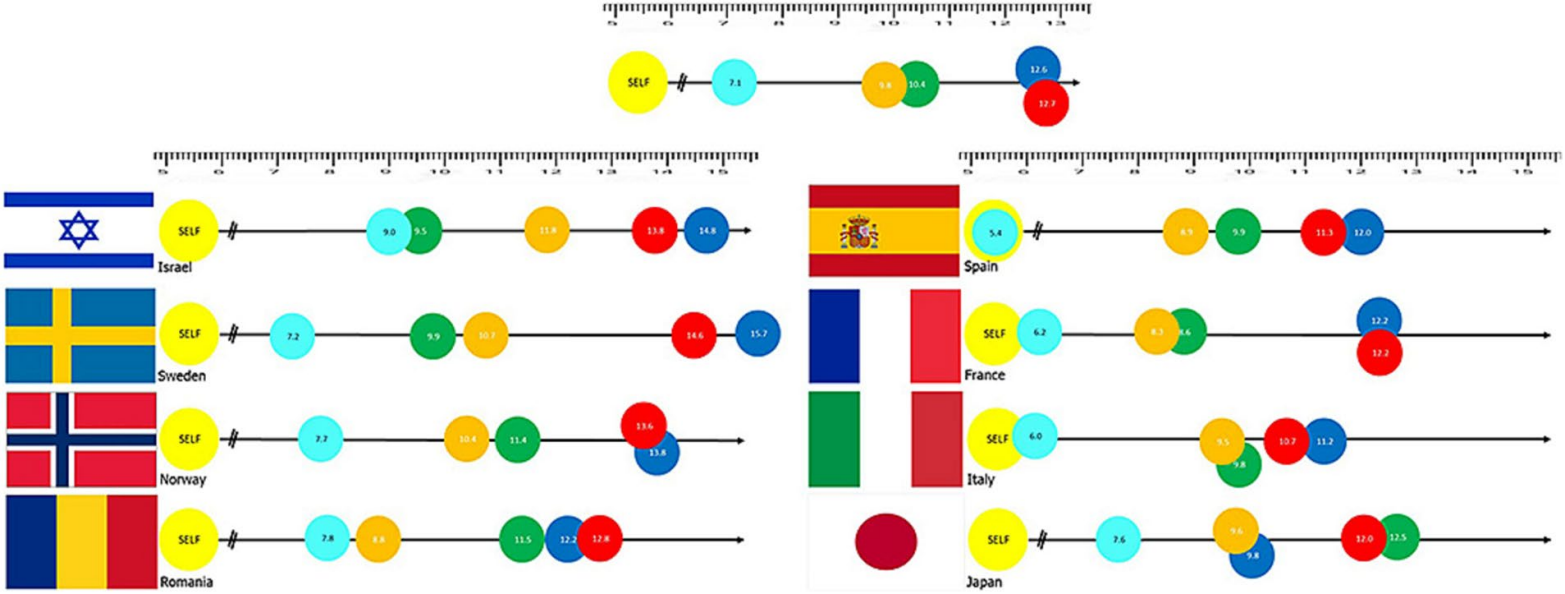
The iPrism tool. Results of the iPRISM tool assessing risk awareness through distances assigned by participants between themselves (yellow “SELF” disk) and specific risk objects [Light blue: Panldemics, Orange: Critical infrastructure fail (water, energy), Green: Social disruption (e.g., war), Blue: Natural Hazard (e.g., earthquakes), and Red: Extreme weather]. Taken from Bodas et al. (2022).

For Population number 1, the questionnaire was based on a Likert scale ranging from 1 (not at all aware) to 5 (extremely aware) to assess the risk perceptions of the members of the emergency services. For Population number 2, we used a designated tool which is the digital version of the Pictorial Representation of Illness and Self-Measure (iPRISM) tool, developed by Büchi and Sensky (1999). This tool was initially used to graphically assess the perceived possibility of suffering from an illness, but it has since been demonstrated that it can also be used for a wide range of applications (Bodas et al., 2022). In this study, the iPRISM tool, shown in Figure 1, was used to rapidly assess the perceived level of risk for each type of risk. The iPRISM tool showed the participants a digital white rectangular board with a fixed yellow disk at the bottom right corner. The participants were instructed that the yellow disk represented themselves and the whiteboard represented their life at that moment. Moreover, the tool provided five colored disks, each representing a different type of risk. Participants were asked to place the colored disks on the whiteboard, relative to the yellow disk, based on their risk perception, meaning that if the colored disk is placed far from the yellow disk, the perceived risk is low, and vice versa. The results obtained from iPRISM are the distance, in centimeters, between the colored disks and the yellow disk. Distance measures ranged between 0 and 26 cm, with smaller values representing higher risk perception levels. The main advantage of this tool is that it enables us to assess the risk perception visually, in a universal language, facilitating its understanding across different cultures.

The questionnaires were tailored to the specific needs and levels of understanding of the two target populations, and thus the questionnaire for the population was based on visual representation of the five risks, while the first responders and authorities were asked directly to rank the varied risks (the next sub-section includes more details about the used tools).

### Data collection

The responses of Population number 1 were collected using the SurveyMonkey^1^ web service. We used the Snowball sampling technique to disseminate the survey and to reach the maximal number of responders. Snowball sampling was the most appropriate sampling method considering the specific characteristics of the targeted survey respondents, and thus responders were asked to share the questionnaire with their colleagues. The questionnaire was distributed by the authors of this article and other partners in a consortium through personal and professional connections.

Concerning Population number 2, we contracted the iPanel company for the data collection task. iPanel is an Israeli company that provides online data collection services since 2006. The company subcontracted local vendors in the other participating countries to gather the needed data. Due to the typical characteristics of Population number 2 and the expertise of iPanel company in online polling, utilizing them for this task proved to be efficient. However, due to the specific nature of the respondents in Population number 1, it was not possible to use an internet panel company, and thus a direct approach to those respondents was required.

Both surveys were available in seven languages corresponding to the official language of the participants’ countries, namely: French, Hebrew, Italian, Norwegian, Romanian, Spanish, and Swedish. Due to the high scope of the survey, we limited the survey language for the official language in each country, excluding minorities’ original languages. While this can create a potential bias in the results. However, as mentioned before, the use of the iPrism tool, as a visual one, narrowed this potential bias.

### Statistical analysis

Descriptive statistics were used to describe the characteristics of the sample for each country. In order to compare the two groups (the first responders versus the population) first, a combination of the two scales was needed. The scale provided to the first responders ranked from 1 to 5 where the higher the number, the higher the risk perception level. Meanwhile, the risk perception of the population was measured by iPRISM. In this case, the scale ranged from 0 to 26 and the direction of the scale was the opposite, i.e., the higher the number, the lower the risk perception level. Therefore, we standardized the two scales through these 3 steps:

1. Transforming the range of answers of First Responders from 1 to 5 to 0–4 (being 1 = 0, 2 = 1, 3 = 2, 4 = 3, 5 = 4).
2. Dividing the scale of Population (Originally scaled from 0 to 26) by 6.5 to be scaled from 0 to 4.
3. Transforming the population scale (After dividing by 6.5) to the opposite direction, using the function (4-x) so that the direction of the scale will be the same as the one of the First Responders: the higher the number, the higher the level of risk perception.

After standardizing the scales, the different perceptions of risks were analyzed using univariate analysis ANOVA with 3 effects: Group effect (i.e., differences between first responders versus the population); Country effect (i.e., differences between the 7 countries); Interaction effect group & country (i.e., we examined whether the two independent variables together [group and country] simultaneously affected the risk perception to a greater extent than the sum of their parts). The results of the 3 effects are presented with *F* value (the ratio between the two variances) value of p (level of significance) and the effect size by partial eta square (ηp2) below each graph. The Bonferroni test (Bland and Altman, 1995) was used for multiple comparisons between countries. The results of the Bonferroni test are presented below each graph in a matrix table using the value of p. All statistical analyses were performed using SPSS software version 25. *p*-values lower than 0.05 were considered to be statistically significant.

Both surveys controlled for demographic variables, as described in Table 1. Furthermore, in the first responders survey, we also incorporated professional variables, such as the individual’s role within the organization and the organizational type. Due to variations in sampling methods and the populations reached by each survey, we did not include these variables in the combined analysis. Nevertheless, separate analyses conducted for each sample, which have been presented in other publications, revealed minimal effects of these control variables (Bodas et al., 2022; Elkady et al., 2022).

**Table 1:** Descriptive statistics of the study population.

	Israel (P) (*n* = 731) (FR) (*n* = 224)	Sweden (P) (*n* = 521) (FR) (*n* = 17)	Norway (P) (*n* = 686) (FR) (*n* = 186)	Romania (P) (*n* = 691) (FR) (*n* = 189)	Spain (P) (*n* = 675) (FR) (*n* = 173)	France (P) (*n* = 527) (FR) (*n* = 24)	Italy (P) (*n* = 536) (FR) (*n* = 36)
Group *(%)*							
Population	68.9	96.7	72.9	72.4	74.4	95.4	93.3
First responders^*^	31.1	3.3	27.1	27.6	25.6	4.6	6.7
Age group *(%)*							
20–35	37.7	39.9	33.1	38.8	33.8	39.0	39.4
36–50	30.4	31.2	30.7	43.3	40.4	34.9	35.4
51–65	26.4	27.6	31.3	17.9	25.6	24.0	22.8
66+	5.3	1.4	4.8	0	0.2	2.2	2.5
Gender *(%)*							
Male	49.8	48.4	44.2	41.8	43.3	47.7	47.2
Female	51.1	51.6	55.8	58.2	56.7	52.3	52.8

*Breakdown of the first responders is delineated in Appendix A.

## Results

The study was conducted among samples of both the population and first responders in six European countries as well as in Israel. The samples in each country included at least 500 respondents from the population, while the samples of the first responders ranged from 227 in Israel to 17 in Sweden (Elkady et al., 2022). In the overall sample (including both first responders and the public) 38% were in the age group 20–35, 35% were in the age group 35–50, 25% were in the age group 51–65, and 2% were at the age group 66 and above. A slightly higher percentage of women compared to men responded to the surveys. See Table 1.

### Ranks of the perceived risks

The average scores of the perceived risks were calculated to identify differences in risk perceptions between the population and the first responders in each of the seven countries as well as between the two groups in the varied countries. Based on the average scores, we ranked the risks from 1 to 5, where 1 represents the highest risk perception and 5 represents the lowest. See Table 2.

**Table 2:** Ranks of the five perceived risks, according to the two groups (population vs. first responders in the seven countries), 1 being the most severe risk and 5 being the least severe risk.

Country	Group	Extreme weather	Naturerelated events	Social disruptions	Critical services dependencies	Pandemics
Israel	Population	5	4	2	3	1
	First responders	5	3	2	4	1
	The gap	**0**	**1**	**0**	**−1**	**0**
Sweden	population	4	5	2	3	1
	first responders	2	3	2	4	1
	The gap	**2**	**2**	**0**	**−1**	**0**
Norway	population	4	5	3	2	1
	First responders	3	5	4	2	1
	The gap	**1**	**0**	**−1**	**0**	**0**
Romania	population	5	4	3	2	1
	first responders	5	2	4	3	1
	The gap	**0**	**2**	**−1**	**−1**	**0**
Spain	population	4	5	3	2	1
	first responders	2	5	3	4	1
	The gap	**2**	**0**	**0**	**−2**	**0**
France	population	4	5	3	2	1
	First responders	3	4	1	2	1
	The gap	**1**	**1**	**2**	**0**	**0**
Italy	population	4	5	3	2	1
	First responders	1	2	3	4	1
	The gap	−3	−3	−1	−2	0

As can be expected, considering that the data collection was conducted during the COVID−19 pandemic, both groups of respondents in all seven countries ranked pandemics as the highest risk. Social disruptions were ranked as the second highest risk by both the populations and the first responders from Israel and Sweden. Conversely, critical services dependencies were reported as the second highest risk by both the population and the first responders from Norway and France, and by the populations (but not the first responders) from Romania, Spain, and Italy. Extreme weather was reported as the lowest risk by both the population and the first responders from Romania and Israel. In contrast, nature-related events were perceived as the lowest risk by the population and first respondents from (Spain, Norway, and France), and by the population (but not the first responders) from Sweden and Italy. The biggest diversities between population and first responders within the respective countries were identified in Italy (concerning extreme weather and nature-related events), while Norway, Spain, and Israel respectively, presented similar perceptions among the population and the first responders in three out of the five investigated risks.

### Risk perceptions concerning extreme weather

**Figure 2:**
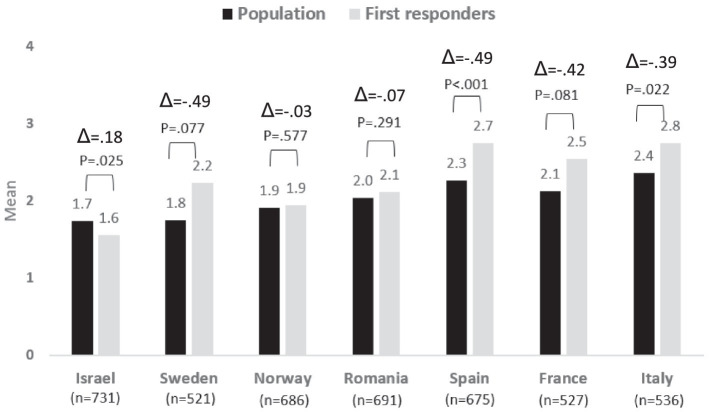
Risk perception regarding Extreme Weather − differences between country and group: population vs. first responders.

The risk perceptions of the population, compared to the first responders, concerning extreme weather were lower in most countries, except for Israel and Norway, though these differences were found to be significant only in Spain, Israel, and Italy. In Norway, the extreme weather was perceived similarly by the population and the first responders, while in Israel, the population perceived this risk as significantly more severe than perceived by the first responders. A comparison of the risk perceptions among the different countries presents that extreme weather is perceived by Spanish, French, and Italian respondents as a higher risk compared to Romanian, Swedish, Norwegian, and Israeli respondents. See Figure 2.

Regarding the existing differences between the first responders’ and population’s risk awareness levels, we can see that the bigger difference exists in Spain and Sweden followed by France and Italy. On the contrary, Norway is the country with a lower difference among the two populations, followed by Romania.

### Risk perceptions concerning nature related events

The risk perceptions of the population, compared to the first responders, concerning nature-related events were lower in most countries, except for Norway, where the population perceived these risks as significantly higher than those that were reported by the first responders. Significant differences between the two populations were also identified in both Romania and Italy, where as noted, the first responders perceived these risks as more challenging than the populations.

**Figure 3:**
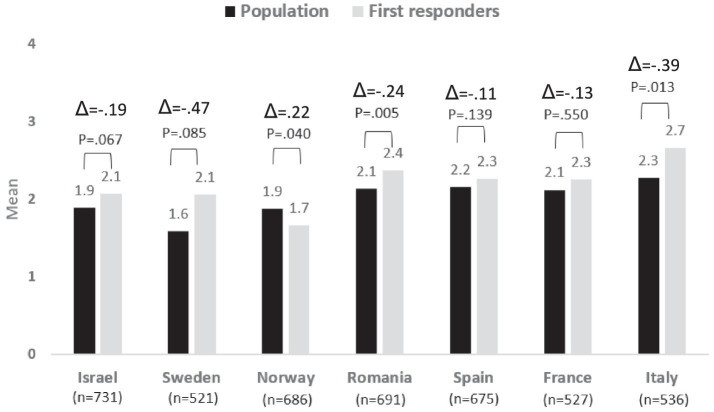
Risk perception regarding Nature Related Events − differences between country and group: population vs. first responders.

A comparison of the risk perceptions among the different countries presents no significant differences in risk perceptions concerning nature-related events between responders from Italy, Spain, France, and Romania. The risk perceptions among respondents from these four countries are significantly higher than those of respondents from Norway, Sweden, and Israel. See Figure 3.

Regarding the differences in risk awareness between the two populations, Sweden is the country that presents the highest difference between the first responders and the population, and Italy is the second one. Conversely, Spain is the country where the difference between the two populations is the lowest followed by France.

### Risk perceptions concerning social disruptions

The risk perceptions of the population, compared to the first responders, concerning social disruptions were found to be higher in Israel, Sweden, and Norway, though the variance was found to be significant between the two populations only in Israel and Norway. Similar levels of risk perceptions were found among both the population and the first responders in Romania, France, and Italy, whereas the population in Spain perceived the risk of social disruptions as somewhat less severe (significantly) compared to the first responders.

**Figure 4:**
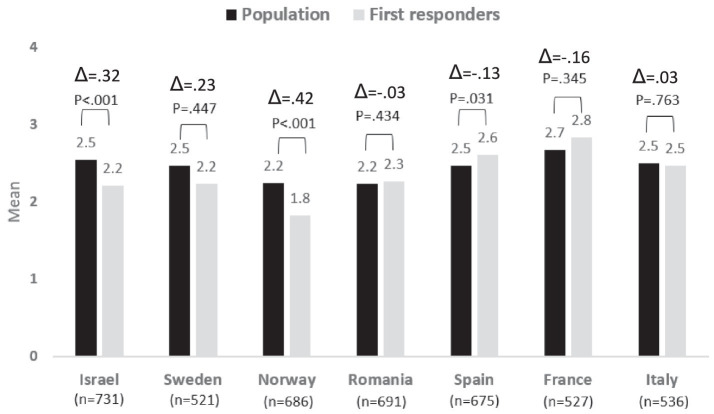
Risk perception regarding Social Disruption − differences between country and group: population vs. first responders.

A comparison of the risk perceptions among the different countries shows that French respondents perceived this risk as significantly higher compared to the other six countries. No significant risk perceptions were found among respondents from Italy, Israel, Sweden, and Spain. The levels of risk perceptions among the Romanian respondents differed significantly from all other countries, but Norway and vice versa; the risk perceptions among the Norwegian respondents differed significantly from all other countries, but Romania. See Figure 4.

### Risk perceptions concerning critical services dependencies

The risk perceptions of the population, compared to the first responders, concerning critical services dependencies, were found to be higher in all countries, except in France (but the difference between the two groups of population and first responders was significant only in Norway, Romania, and Italy).

**Figure 5:**
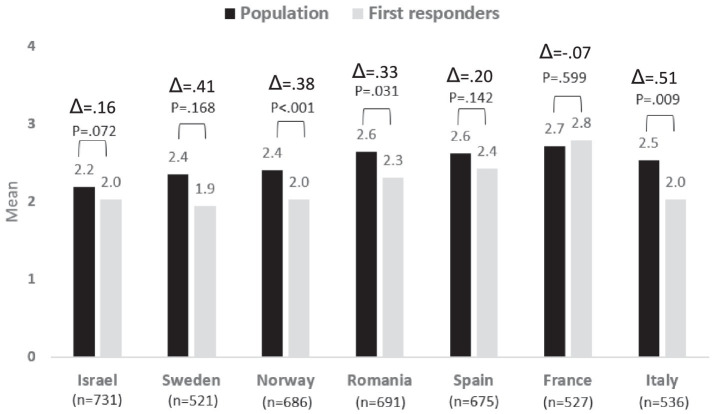
Risk perception regarding Critical Services Dependencies − differences between country and group: population vs. first responders.

Similar to what was found concerning social disruptions, French respondents perceived this risk as higher compared to the other six countries, though the difference was found to be significant only compared to Israel, Italy, Norway, and Sweden. Romania also perceived this threat as more severe compared to all other countries but France, but the differences were found to be significant only in relation to the risk perception of the populations in Israel, Sweden, and Norway. See Figure 5.

In this type of disaster, the differences in risk awareness levels for both populations are quite high, with Italy being the one with the highest difference. Sweden and Norway are the next ones followed by Romania. France is the country that presents the lowest difference in the risk awareness levels of the two populations.

### Risk perceptions concerning pandemics

The risk perceptions of the population, compared to the first responders, concerning pandemics, were found to be higher in all countries, except Spain. The differences between the two groups of population and first responders were significant only in Norway, Romania, and Spain. The risk perception of the population in Spain was significantly lower compared to that of the first responders. Sweden is the country that presents the highest difference in the risk awareness level of the two populations. Norway is the second country with the highest difference and Italy the third. In the three cases, the awareness level of the population is higher than that of the first responders. Conversely, Israel is the country with the lowest difference followed by Spain, although in the opposite direction, the first responders’ risk awareness level is higher than the population’s risk awareness level.

**Figure 6:**
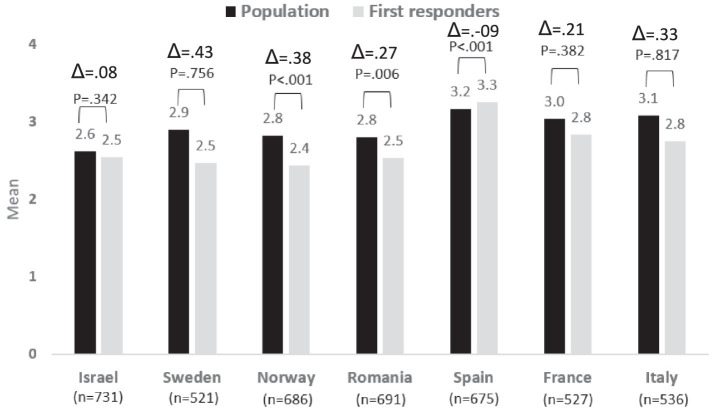
Risk perception regarding Pandemics − differences between country and group: population vs. first responders.

When comparing the risk perceptions of the different countries concerning pandemics, the highest risk perception was found among the sample from Spain, regarding both the first responders and the population. This risk perception was found to be significantly higher than the risk perceptions of the respondents from Israel, Sweden, Norway, and Romania (but not significantly different from France and Italy). The average levels of the perceived risk of pandemics were similar among the first responders from Israel, Sweden, Norway, and Romania, but the risk perceptions of the populations in those countries varied, resulting in significant differences in the overall samples only between Israel and Sweden as well as between Norway and Sweden. See Figure 6.

### National differences

**Figure 7:**
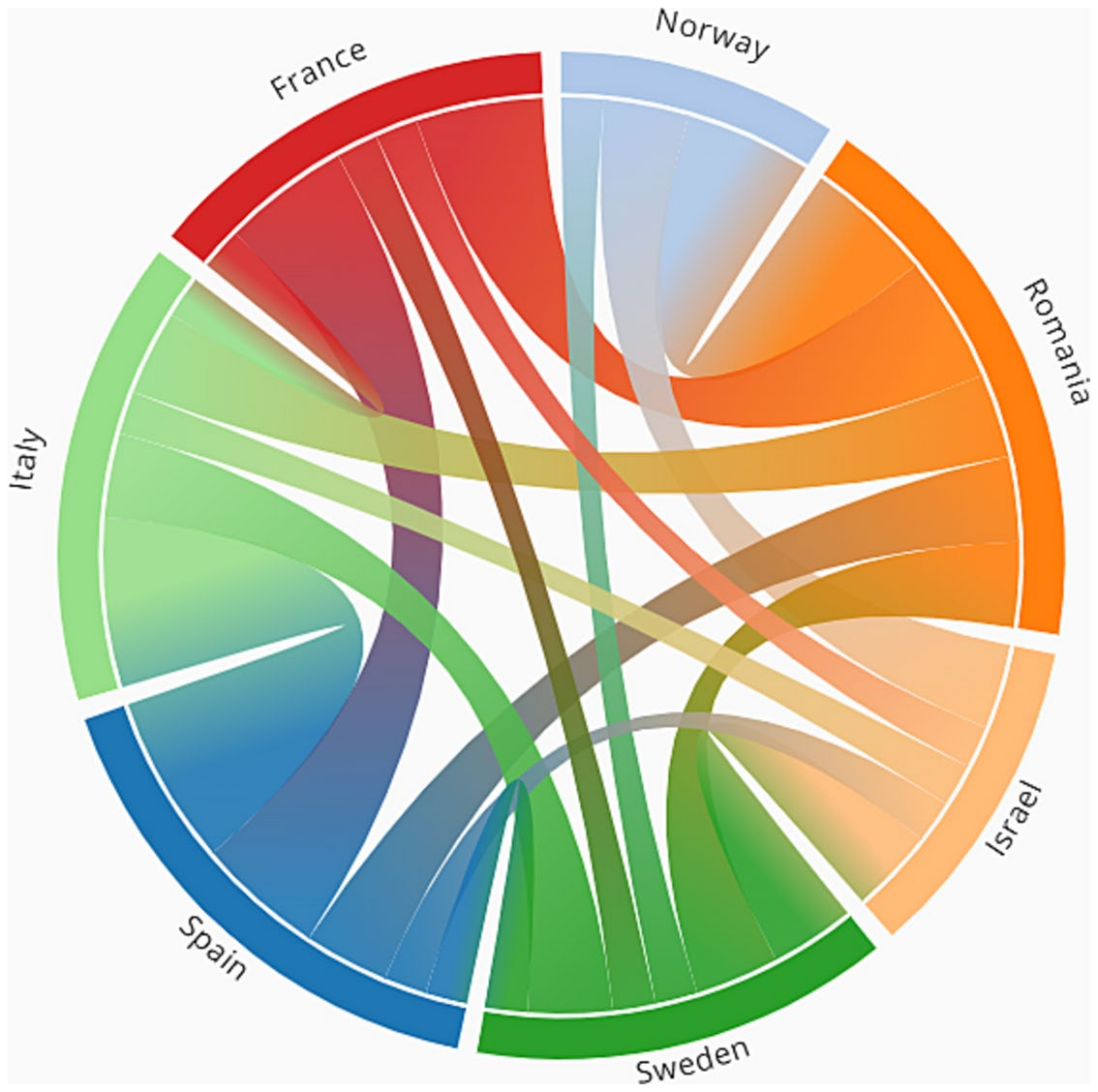
The similarities between the countries in terms of risk perceptions.

Following the results regarding the national differences in risk perceptions of both first responders and the general public, Figure 7 presents the similarities and differences between the countries regarding their risk perceptions.

In this figure, each of the countries is represented as a node or as part of the outer layout of the circle. The arcs between the countries represent the similarity between the countries in risk perception. For example, an edge between Israel and Norway means that Israel and Norway share at least one commonality in one of the categories of risk perceptions. The commonality is defined when there is no significant difference in a risk perception category (e.g., in the case of Israel and Norway, there were no differences between the countries concerning Natural Related Events). The number of categories of risk perceptions with commonalities between the countries defines the thickness of the edges. For example, if there is a commonality in one category then the edge thickness is 1, if there are in two categories, then it is 2, etc. The figure shows that Southern Europe countries such as Spain and Italy, share many commonalities across all risk perception categories. In addition, despite being part of Scandinavia, Norway and Sweden share fewer commonalities than Southern European countries or even compared to their commonalities with Romania. Israel, on the other hand, despite its geographical distance, has some commonalities with Norway and Sweden.

## Discussion

As countries are prone to different types of risks, they are expected to present varied risk perceptions. Nonetheless, as some risks are common to a wide range of countries, such as pandemics in general, and COVID−19 in particular, it is essential to understand why they may differ in their risk perceptions of those similar hazards. Furthermore, as displayed in the current study, variabilities may frequently exist within each society when comparing the risk perceptions of first responders with those of the general public. Therefore, we will elaborate on the possible reasons for such differences and their importance.

Our findings resonate with the established concept of the risk perception paradox. While our study highlights varied risk perceptions across different demographics and regions, it’s essential to understand that high risk perception does not always translate to proactive preparedness or mitigation actions. As elucidated in the literature, individuals might be fully aware of the risks but might choose to accept them, especially if the perceived benefits overshadow the potential threats (Wachinger et al., 2013). In other instances, the sense of agency might be lacking, leading individuals to transfer the onus of action to others (Shapira et al., 2018). Economic constraints and personal conditions can also hinder individuals from taking preventive measures (Wachinger et al., 2013). This understanding underscores the importance of not just raising awareness but also empowering individuals with the means and motivation to act upon their risk perceptions.

The current study identified commonalities and variabilities in risk perceptions among the general population compared to first responders within and between different countries. The most significant outcome of the study was that the public and first responders in some countries ranked all of the investigated risks (extreme weather, nature-related events, social disruptions, and critical services dependencies) differently except for pandemics, and they also differed in the respective levels of their risk perception. These differences in risk perceptions were found between the two groups both within the investigated countries, as well as between the countries.

As the study occurred during the COVID-19 pandemic, it is not surprising that the only consensus between the public and first responders within and between all the countries was ranking pandemics as their highest concern. Nevertheless, the difference in the perceived risk between the population versus the first responders was relatively (and significantly) high in both Norway and Romania. A potential explanation for this difference within the Romanian society, most especially concerning the civilian population, may be the effect of the strict lockdowns, which led to a sharp increase in the risk perception of the public (Lindner et al., 2022). In contrast, the first responders believed that such measures decrease the probability of negative impacts (i.e., limit exposure and spread of the virus), which may have led them to optimism bias (Druică et al., 2020). Similarly, Norway had the second highest rate of confirmed cases *per capita*, after Italy, especially in the earlier stages of the pandemic. Accordingly, this may have affected the risk perception of the public, resulting in their belief that the healthcare system is inexperienced in treating pandemics, leading them to a higher risk perception compared to first responders (Zickfeld et al., 2020).

Regarding Social Disruptions, one possible explanation for the different ranking of risks within France, between the general population and first responders, could be related to the effect of two major social disruption events that occurred in Paris in 2019, highly impacting the society. Substantial criticism was voiced by the public regarding the management by the municipality and emergency organizations of the explosion event in January 2019 (Bürkli, 2020) and the fire in the Notre-Dame cathedral in April, 2019 (Pett, 2019). Previous studies have shown that social disruptions may highly impact first responders over time, even more than they affect the public (Klimley et al., 2018; Motreff et al., 2020). Therefore, it could explain why in the case of France these events led the first responders to rank the social disruptions risk higher than the general population.

The higher risk perception concerning social disruption within the general population in Israel, compared to the first responders, could be a result of the threat of terror attacks. Terror events in Israel are frequent (Hirsch-Hoefler et al., 2016). While first responders are trained to handle and respond to terror attacks, the general population may not have the same level of training and experience (Ashkenazi and Hunt, 2019). Additionally, first responders have a better understanding of the measures that are in place to protect them and the public, which can reduce their perception of the risk (Geiger, 2016). In Norway, the higher risk perceptions that were identified among the general population, compared to the first responders, could be related to the higher media coverage of social disruptions that result from the surge of refugees, echoing such issues in the public’s agenda (Hagelund, 2020). In contrast, the higher risk perception among the Spanish first responders, compared to the general public, could be derived from social disruption events such as cyber-terror. The public is not always aware of such attacks, hence perceives the risk as less severe, compared to the first responders that are more exposed to it (Muthuppalaniappan and Stevenson, 2021).

Regarding the ranking of critical services dependencies risks, in all countries but France, the first responders presented lower levels of perceived risks, compared to their respective populations. There are several reasons for this phenomenon. Firstly, emergency responders tend to prioritize addressing risks that pose a direct threat to life and injury over those that disrupt daily societal activities. For instance, they may prioritize extinguishing a fire over addressing disruption in a major road or water network, even though such disruptions can cause hardship for community members. Secondly, many critical infrastructures in Europe are operated by the private sector (Renda and Hammerli, 2010), making companies responsible for handling issues with these systems. Emergency responders may only become involved if the event has a fatal impact. Meanwhile, citizens experience disruptions from the outset. Thirdly, emergency responders often operate in a compartmentalized manner (Loggins et al., 2019), which can affect their priorities. They may prioritize fixing disruptions in the systems for which they are responsible for, without considering the interdependencies between different infrastructures which impact the citizens. More specifically in Spain and Italy this phenomenon aligns with the findings of previous studies in both Spain (Labaka et al., 2016), and Italy (Rehak et al., 2022), which claimed that the implementation of critical infrastructure resilience frameworks is lacking. According to O'Sullivan et al. (2012), a lack of resilience frameworks may imply that the first responders do not appropriately perceive the actual risks, and thus are oblivious to the higher risk perceptions of the public. In Romania, the critical service’s dependencies have been a major focus in recent decades, which most probably contributed to the raised awareness of the public (Gheorghiu et al., 2013; Ozunu et al., 2021). Similarly, as this topic was extensively discussed in the Norwegian media, a similar tendency was found in Norway (Hagelund, 2020).

In the category of extreme weather events, the higher rankings of first responders, compared to the general population in Sweden, Spain, and Italy, could be related to the emergency preparedness programs for weather events in those countries. Previous studies already displayed a global trend of elevated risk perceptions of emergency authorities, regarding the effect of extreme weather events, in particular in those countries (Sovacool, et al., 2018; Perera et al., 2020). However, as extreme weather events are less frequent than other types of disasters, the general population’s risk perceptions may be less affected than the first responders who are trained for such events (Zhang and Maroulis, 2021).

Similar claims could also be made regarding the higher rankings of nature-related events in Sweden, Romania, and Italy. In the case of Italy, events such as the L’Aquila earthquake can explain the higher risk perception of the first responders, as they are involved as vital bodies in such events (Alexander, 2010). According to Paleari (2018), Italy is exposed to a significant number of natural risks. This may lead the Italian government to earmark financial resources to risk prevention and mitigation, resonating such risks in the eyes of emergency professionals, and among them first responders, more than the public. Similarly, Armaş (2006) portrays a possible explanation regarding Romania, with cities such as Bucharest (with the highest seismic risks in the world), leading to higher risk perceptions among first responders, but poor education of the population regarding those risks, which, according to Appleby-Arnold et al. (2021), can lead to a low perceived threat among the population, that may be oblivious to the danger. Furthermore, the literature presents high evidence of actions implemented in Romania, by emergency organizations in general and first responders in particular, to study and improve the risk management of such events, including raising the risk awareness of emergency agencies (Mara and Vlad, 2009; Ozunu et al., 2011; Meltzer et al., 2018).

The fact that the general population in Norway had higher risk perceptions regarding nature-related events, compared to first responders, can be explained by their beliefs about the effects of climate change on nature-related disasters. Hanssen-Bauer et al. (2009) previously claimed that climate change could have many positive effects on Norway, compared to other types of adversities. However, while this may lead to lower risk perceptions among first responders, who are trained and more familiar with the risks of climate change, Lujala et al. (2015) showed reverse effects among the public – who tend to be more concerned about climate change. As nature-related disasters may be more complex to understand and to be anticipated by the general population, their risk perceptions may be higher.

Beyond the variabilities that were found in the study between the general population’s risk perception and first responders, within each country, this study highlighted differences between the countries. This variability could be derived from diverse social and cultural characteristics that differentiate between the societies, even when they are located in similar geographic locations or have been exposed to comparable types of adversities (Viklund, 2003; Drakos and Müller, 2014).

For example, the higher significant gap (0.38) in Norway, compared to Romania (0.27) concerning pandemics could be a direct result of the effect of COVID-19, that, had a greater effect in Norway (Zickfeld et al., 2020).

Concerning extreme weather events, the difference between Spain and Italy, where first responders had higher risk perceptions, to Israel, where the general population had a slightly higher score of risk perceptions, could be derived from the extreme weather resilience frameworks that operate in those countries, compared to Israel (Green et al., 2013; Hudson et al., 2020; Finzi et al., 2021).

The frequent nature-related disasters and their severity in Italy (Alexander, 2010; Paleari, 2018) could explain the higher gap that was presented between the first responders and the public (0.39), compared to the gap found in Romania (0.24).

The larger gap in Norway, between first responders and the public concerning social disruptions, compared to Israel, could be a result of the varied types of emergencies that explain these gaps within each country. In Israel, the main risk is derived from terror events, which are perceived to pose a greater danger to lives (Hirsch-Hoefler et al., 2016), compared to the complexities that result from the absorption of refugees in Norway (Hagelund, 2020).

Concerning critical services dependencies, the larger gap that was found between the general population and first responders in Italy, compared to Romania and Norway, emphasizes the importance of developing CI resilience emergency frameworks (Labaka et al., 2016). This gap presents how the lack of such frameworks enlarges the difference between the public and first responders’ perceptions, compared to countries such as Romania and Norway, in which this topic receives more attention among emergency organizations (Gheorghiu et al., 2013; Hagelund, 2020; Ozunu et al., 2021).

The differences within countries, and the variability in the gaps between the countries, in the risk perceptions of the general population, compared to first responders, support previous studies which claimed that first responders, as a specific professional group, differ from the general public regarding specific demographic characteristics (Brown et al., 2021; Kollmann et al., 2022; Shah et al., 2022) or personality traits (Al-Dahash et al., 2022). The contribution of the discussion made in this study is in connecting these gaps with additional possible explanations, such as different policies in the various countries, the relative focus given for each type of emergency, and the frequency of events.

The differences in risk perceptions between the population and the first responders may lead to challenges in the public’s adherence to the directives issued during adversities by the authorities and first responders. Behavioral models, such as the Health Belief Model (Kamran et al., 2021) and Protection Motivation Theory (Gumasing et al., 2022), predict an association between risk perception, compliance, and behavior. Thus, the gaps identified may affect compliance with the authorities’ or first responders’ instructions concerning the needed protective behavior, in preparation for or during the materialization of hazards (Barr et al., 2008; Jacobs et al., 2010).

Cases in which the public has a higher risk perception may result in two contrasting phenomena. First, the first responders may not be sufficiently sensitive to the risk perceptions level of the public as well as to their needs and expectations concerning those risks, given that they perceive those risks as being less severe (Lohiniva et al., 2020). Second, the overestimation of a particular risk by the general public may lead to a lower preparedness for a more critical risk, that will be ignored (Hengen and Alpers, 2019; Abel et al., 2021). Furthermore, these different perceptions may lead to a growing rift between the public and the first responders, derived from their respective frustration caused by the different levels of risk assessments, as was strongly shown during the COVID-19 pandemic (Bruinen de Bruin et al., 2020; Peleg et al., 2021; Scandurra et al., 2021).

Regarding the first responders, their risk perceptions may affect how they communicate the risk to the population. For example, lower risk perception of the public, compared to risk perceptions of first responders, might result in less compliance with the recommendations – derived from the disbelief of the public that they are necessary (Drury et al., 2019; Cairney and Wellstead, 2021). In contrast, higher risk perception among the public, compared to those of first responders, may create a feeling of being neglected (Simione and Gnagnarella, 2020). For example, make them feel that they are in danger and the official authorities are not providing any assistance, while the first responders perceive this risk as lower than other risks, and thus do not invest wide efforts in protecting the public.

Diversities in the risk perception between countries can result in different adoption of protective measures; for example, that might affect the development of the pandemic’s global management. Therefore, another significant contribution of this study is highlighting those worldwide diversities.

Another important contribution of this study is the influence of context on the existing differences between the general population and first responders. This might be due to a lack of trust among the authorities and first responders, different levels of preparedness, different policies regarding risk communication, and more.

## Limitations

This study has several limitations. First, this study is based on integrating two surveys with different sampling methods. The general population survey was limited to a sample size of 500 in each country, using random sampling. For some countries, this sample size is adequate, while in others, it may cause difficulties in representing the variety of the population (Bodas et al., 2022). The second study used non-random convenience sampling, resulting in diversities in each country’s sample size. Therefore, the conclusions from this study, especially regarding first responders, should be generalized with caution. Second, the national comparison of the study is based on seven specific countries. Factors such as cultural characteristics and geographical environments may complicate the generalization of the commonalities and diversities beyond the sampled countries. It also should be taken into consideration that the data was collected during the COVID-19 pandemic. Therefore, potential limitations or biases can affect the responses of the first responders and the general population, as a result of the pandemic or that their risk perception changed since the availability of COVID-19 vaccinations.

## Conclusion

The findings of this study offer significant insights for policymakers and emergency response planners across the countries surveyed. This study shows that although there are some commonalities in risk perceptions among varied countries, there are even more critical diversities both between countries but also among first responders and the general public, within and between countries. Such diversities present challenges in the communication of hazards by authorities and first responders to the public. The observed variations in risk perceptions between the general population and first responders emphasize the need for tailored communication strategies for different groups. Policymakers should consider these differences when designing public awareness campaigns, ensuring that messages resonate with the target audience’s unique perspectives.

The differences in the risk perceptions among emergency responders and the populations may impede the implementation of different policies and plans set by the first responders and authorities, as citizens may have different priorities according to their risk perceptions. Furthermore, the ranking of perceived risks can guide resource allocation, prioritizing areas deemed as higher risks by both the public and first responders. The disparities in risk perceptions between countries also suggest the importance of context-specific strategies, taking into account cultural, historical, and socio-economic factors. Such misalignment would require the first responders to be more actively involved in the protection of civilians as they may be unprepared to handle crises due to their lack of awareness. In line with this constraint, future research is needed to investigate how to bridge the gap in the risk perception of both types of populations, to avoid the previously mentioned challenges.

Furthermore, the difference in the risk perceptions among countries reinforces the strategic approach that resilience is contextualized and efforts to enhance it should be tailor-made, considering the specific characteristics of each society, as no one policy ‘fits it all’.

Lastly, the study underscores the importance of continuous training for first responders, ensuring they are well-equipped to address the most pressing risks in their respective regions.

## Data availability statement

The datasets presented in this article are not readily available because the data collected in this study is not publicly published due to the requirements set by the Ethics Committee that approved the study. Existing analyzed anonymized data will be made available to researchers upon request. Requests to access the datasets should be directed to BA, adini@tauex.tau.ac.il.

## Ethics statement

The studies involving humans were approved by Tel Aviv University Ethics Committee. The studies were conducted in accordance with the local legislation and institutional requirements. The participants provided their written informed consent to participate in this study.

## Author contributions

BA and LL conceptualized the study. NS and SE collected the data and analyzed the findings, and wrote the first draft. MS conducted the statistical analysis. All authors reviewed the manuscript and modified as needed.

## Footnotes

^1^https://www.surveymonkey.com/
